# Automated workflows using Quantitative Colour Pattern Analysis (QCPA): a guide to batch processing and downstream data analysis

**DOI:** 10.1007/s10682-024-10291-7

**Published:** 2024-03-16

**Authors:** Cedric P. van den Berg, Nicholas D. Condon, Cara Conradsen, Thomas E. White, Karen L. Cheney

**Affiliations:** 1 School of the Environment, The University of Queensland, Brisbane, QLD 4072 Australia; 2 School of Life and Environmental Sciences, The University of Sydney, Sydney, NSW 2050 Australia; 3 School of Biological Sciences, University of Bristol, Bristol, BS8 1TQ UK; 4 Institute for Molecular Bioscience, The University of Queensland, Brisbane, QLD 4072 Australia

**Keywords:** ImageJ, Java, R software, Automation, Animal colouration, Visual ecology

## Abstract

Animal and plant colouration presents a striking dimension of phenotypic variation, the study of which has driven general advances in ecology, evolution, and animal behaviour. Quantitative Colour Pattern Analysis (QCPA) is a dynamic framework for analysing colour patterns through the eyes of non-human observers. However, its extensive array of user-defined image processing and analysis tools means image analysis is often time-consuming. This hinders the full use of analytical power provided by QCPA and its application to large datasets. Here, we offer a robust and comprehensive batch script, allowing users to automate many QCPA workflows. We also provide a complimentary set of useful R scripts for downstream data extraction and analysis. The presented batch processing extension will empower users to further utilise the analytical power of QCPA and facilitate the development of customised semi-automated workflows. Such quantitatively scaled workflows are crucial for exploring colour pattern spaces and developing ever-richer frameworks for analysing organismal colouration accounting for visual perception in animals other than humans. These advances will, in turn, facilitate testing hypotheses on the function and evolution of vision and signals at quantitative and qualitative scales, which are otherwise computationally unfeasible.

**Supplementary Information:**

The online version contains supplementary material available at 10.1007/s10682-024-10291-7.

## Introduction

Understanding the perception of visual information by non-human observers is crucial to studying the ecology and evolution of animal and plant colouration. The last two decades have seen the creation and widespread adaptation of tools and methods that allow researchers to simulate or approximate aspects of animal vision, such as colour contrast perception (e.g. Endler and Mielke 2005; Gawryszewski 2018; Kemp et al. 2015; Renoult et al. 2017; Vorobyev and Osorio 1998) and spatial vision (Godfrey et al. 1987; Caves and Johnsen 2018). These advances coincide with the steady development of colour pattern analyses (e.g. Chan et al. 2018; Endler 2012; Stoddard et al. 2014; Van Belleghem et al. 2018; van den Berg et al. 2020b) and their integration into increasingly comprehensive collections of tools and functions across software platforms such as pavo (Maia et al. 2019) in R (R Core Team 2021) or the Multispectral Image Calibration and Analysis toolbox (MICA) (Troscianko and Stevens 2015) in ImageJ (Schneider et al. 2012).

Quantitative Colour Pattern Analysis (QCPA) (van den Berg et al. 2020b) is a recent and powerful addition to this landscape. It provides a dynamic analytical framework for analysing visual scenes through the eyes of ecologically relevant observers and is integrated into the MICA toolbox (Troscianko and Stevens 2015). It allows users to choose preferred tools and analyses uniquely suitable for analysing spatiochromatic information. Briefly, it achieves this by taking full-spectrum images as its input (thereby generating ‘.mspec’ files) before applying a suite of models which can consider the spectral, spatial, and temporal sensation of non-human viewers, to ultimately give image-based and numerical outputs that summarise the structure of visual scenes and stimuli. 

However, preparing and processing calibrated images in the QCPA framework remains tedious. This is due to the time needed to translate calibrated digital images into cone catch images, user-guided input for identifying regions of interest (ROIs), and the subsequent, sequential and repeated application of image processing and analyses. Saving and labelling multiple output files, as well as extracting data for subsequent statistical analysis, also remains a tiresome process. An image needs hours of manual, repetitive work to obtain numerical output from multiple analyses in the QCPA framework. For example, preparing and running the analyses outlined in the worked examples Supplement, and saving and consolidating the numerical output can take anywhere from two to four hours of work per image even with comparably minimalist choices of a two, large viewing distances. Processing a hundred images, means investing several hundred hours of work into manual image analysis, depending on the extent of analysis and the user’s familiarity with the software. As a result, applying the QCPA at quantitative scales, such as analysing large numbers of images at multiple viewing distances, is nearly impossible in its original form. Consequently, most studies using the framework do not consider more than a few dozen individual observations with a limited subset of available image statistics (e.g., Nokelainen et al. 2021; Rodríguez-Morales et al. 2021).

Hundreds, or even thousands, of observations are common in comparative studies of animal colouration (e.g., Alfaro et al. 2019; Hoyal Cuthill et al. 2019; Feldmann et al. 2021; Ronco et al. 2021) and colour pattern functionality (e.g., Rönkä et al. 2020; Yong et al. 2022; Kelley et al. 2023). Considering spatial acuity is also increasingly common practice (e.g. Barnett and Cuthill 2015; Caves et al. 2018, 2023; van den Berg et al. 2023). For example, the long-established understanding of multi-component and multi-modal primary and secondary defences and their adaptive purpose in their sequential deployment along an escalating predation sequence (Endler 1986, 1991) has led to consideration of the visual perception of animal colouration across multiple viewing distances. Furthermore, visual signals have likely adapted to being perceived by multiple signal receivers across different viewing contexts, including varying viewing distances, illumination and visual backgrounds (see Kemp et al. 2023 for review). Therefore, thorough investigations of the adaptive properties of animal colouration warrant the repeated analysis of calibrated images across multiple visual systems, lighting conditions, viewing distances, and different areas of each image, including different body parts of an animal, or the animal and its background. Indeed, obtaining numerical output from multiple colour pattern analyses to obtain a broad and differentiated capture of visual phenotypes can be crucial and is a core capacity of QCPA.

Ecologically relevant descriptors of animal colour patterning cannot always be pre-determined without artificially narrowing the dimensionality of colour pattern space before colour pattern analysis. However, this (or omitting the reporting of alternative conducted analyses) is often a tempting solution to simplify downstream statistical analysis. Particularly in observational studies or when precise mechanisms underlying selective pressures shaping phenotypic diversity are unknown, it is arguably beneficial to search for and deduce variation in complex animal colouration (or changes in animal behaviour as a consequence thereof) with the use of highly descriptive multi-dimensional colour pattern spaces and appropriate statistical solutions such as dimensionality reduction (for discussion see Stoddard and Osorio 2019; van den Berg et al. 2020b, 2022; Kemp et al. 2023). For example, the identification of, and distinction between, global (i.e., present across the entire animal) and local (i.e. specific colour pattern elements or body parts) of colour pattern properties and their appearance to specific observers in a given context (i.e., viewing distance) is crucial when explaining the ecology and evolution of complex, multicomponent colour pattern phenotypes (e.g. Howse and Allen 1994; Hebets and Papaj 2005; Stevens and Ruxton 2012; Skelhorn and Rowe 2015). Therefore, considering all possibly relevant image statistics and deducing a relevant set of parameters is often the desired approach instead of pre-emptively narrowing down the number of considered image statistics (van den Berg et al. 2022). However, such deductive approaches require adequate statistical solutions and remain an intriguing challenge in using complex colour pattern spaces (Stoddard and Osorio 2019; van den Berg et al. 2020b, 2022). Highly differentiated colour pattern spaces are further valuable for machine learning applications such as species identification (e.g. Šulc et al. 2021; Carlson and Stoddard 2023) or predicting ecological correlates from appearance such as species habitats or secondary defences and propose an intriguing avenue of future research, further highlighting the need for automating—and therefore facilitating—repeated laborious workflows.

We have reviewed nearly 100 peer-reviewed studies and pre-prints (*n* = 98, Table S1) referencing the QCPA framework (excluding theses and published pre-prints). Out of these studies, 27 make use of the QCPA framework for image analysis, of which four use some form of batch scripting. The median number of images analysed in a study using the framework without any known use of batch scripting is 48.5. Among the four studies where we know batch scripting has been employed, the median number of images used rises to 311. This includes two studies using the presented batch script extension and two cases of custom-written but not publicly shared batch scripts, further highlighting the potential for unequal access to batch scripting among researchers using the QCPA framework. This finding further confirms the discrepancy in the scale of image analysis between studies employing QCPA without batch scripting and comparative analyses or analyses considering otherwise large datasets requiring hundreds or thousands of images. Almost 50% (*n* = 13) of all studies using QCPA have analysed multiple viewing distances, visual systems or ROIs in each image, meaning repeated processing of individual images is common among users.

Small sample sizes are problematic as quantifying ecologically relevant effects, such as colour pattern variability in natural populations or analysing behavioural data, requires large sample sizes to provide adequate statistical power. This holds particularly true for a method producing hundreds of image statistics from a single observation, such as the QCPA, where the number of available image statistics easily outnumbers observations (van den Berg et al. 2022). The QCPA framework is, therefore, in urgent need of automation to facilitate its complete application to large datasets.

Here, we present a dynamic batch-processing extension to QCPA, allowing users to apply almost the entire QCPA framework or selected parts to large datasets via a flexible, GUI-guided input. We further provide a set of complementary R scripts to guide novice users in efficiently extracting numerical data from the consecutively stored output files for downstream analysis. The batch-processing extension enables the reduction of the amount of manual work associated with image analysis in QCPA by orders of magnitude (i.e. reducing hundreds or thousands of hours of active manual work to a few dozen), scaling in effectiveness with the magnitude of intended analysis and the number of repeated analyses for different purposes such as different observers or lighting conditions. Furthermore, automating long, tedious and repetitive tasks significantly reduces the chance of errors and facilitates troubleshooting, further saving a significant amount of time in addition to increasing data quality. We describe the functionality of both—the QCPA batch script and complementary R scripts—and provide detailed worked examples. Specifically, the batch script facilitates the analysis of large datasets using multiple colour pattern analyses across multiple visual systems, lighting environments and viewing distances. This is enabled by a suite of dedicated graphical user interfaces allowing users to choose appropriate settings following an easy routine of preparing their dataset for batch analysis, including the specification of regions of interest (ROIs) and a standardised folder structure. Following the initial setup, the batch script applies all chosen processing steps and image analyses to each image while saving the numerical output in a standardised folder structure, allowing easy navigation and downstream analysis. This transforms the laborious use of QCPA into a ‘setup & forget’ user experience that can be run on multple instances, in parallel and without supervision in cases where users are confronted with large datasets warranting thorough, lengthy or repeated analysis. While this script does not provide a complete solution for every possible application of the QCPA, we hope it will offer a viable solution to most. This batch script should also help researchers customise their automated pipelines, stimulating the open exchange of programming solutions via platforms such as open-access publications, open-access platforms such as GitHub or the dedicated user forum for the MICA toolbox (www.empiricalimaging.com).

## QCPA batch script

### Intended use & functionality

The batch script extension is intended for users with pre-existing experience in the manual application of QCPA. Pre-exisiting familiarity with QCPA will greatly facilitate the user’s understanding of the capabilities and limitations of the presented extension to QCPA. The QCPA batch script is intended to be applied for the quantification of spatiochromatic information of an object (i.e. an animal) against its visual background. As such, the script can also be applied to images without an animal or object of interest or an animal or object itself.

The batch script extension enables the following functionality:

1. Preparing large datasets for repeated automated image analysis.
2. Automated image analysis considering multiple illuminants.
3. Automated application of image processing, such as:
  - Acuity corrections across multiple viewing distances.
  - Artefact removal using edge enhancement.
  - Image segmentation.
4. Automated colour pattern analyses, including:
  - Colour Adjacency Analysis (CAA).
  - Visual Contrast Analysis (VCA).
  - Boundary Strength Analysis (BSA).
  - Particle Analysis.
  - Local Edge Intensity Analysis (LEIA).
  - Colour Maps.
  - GabRat.
5. Automated output file generation and storage.
6. Detailed log file generation for reproducibility.

The script allows the user to choose the following analytical outputs individually: (1) Colour Adjacency Analysis (CAA), Visual Contrast Analysis (VCA), Boundary Strength Analysis (BSA) and particle analysis; (2) Local Edge Intensity Analysis (LEIA); (3) Colour Maps; and (4) GabRat. For a detailed discussion of these analyses, please see the original publications and their modifications for QCPA as listed in van den Berg et al. (2020a, b).

Notably, the script requires cone mapping functions derived from calibrated cameras (several standard profiles are included in the MICA toolbox). Currently, it does not permit the use of chart-based cone mapping models. While covering many analyses available in QCPA and the MICA toolbox, the batch script extension does not provide a comprehensive library of automation. However, the script is designed to be easily modified by the user. Such modifications are explicitly invited from the community, and we encourage their open sharing on community platforms in the interest of mutual benefit among researchers in the field.

### Data preparation

Image processing in QCPA is specific to a variety of user choices specifying the properties of the observer’s visual system, the light environment in which a picture was taken as well as the intended target light environment, observer viewing distance, and a variety of parameters and processing choices that vary depending on the desired analyses. The batch script uses three approaches to minimise the need to repeat the number of times such input is required:

1. Pre-defined folder structure.
2. Auxiliary files.
3. Batch processing graphical user interface (Batch GUI).

#### Folder structure

The batch script requires input data to be organised in a specific way. This refers to the naming of files and the folder structure. For example, the script expects individual observations (i.e., animals or objects) to be grouped within an overarching folder (i.e., species or site) and file naming must be uniform. This allows the script to reliably detect individual observations while allowing the user to structure their data according to sites or taxa (see the manual for detailed information and the worked examples for exemplary implementation). The batch script extension provides a folder checker tool to check the correct setup and naming of folders and content in large datasets (see the manual for details).

#### Auxiliary files

The batch script uses the MICA toolbox’s pre-existing approach to associate several files with each image to be analysed. In addition to the already existing need for a folder with the specified ROIs and the corresponding.mspec of each image, the batch script requires two additional text files specifying the rotation of each image and the cone mapping function (see the manual for details). These allow users to standardise the orientation of all images before analysis and permit multiple cone mapping functions within the same dataset (e.g., multiple lighting environments).

#### Batch GUI

The batch script allows the user to specify which of the available analyses they want to conduct and what settings they want to use. The Batch GUI consists of a central interface and a suite of dedicated, tool-specific GUIs activated by user choices. These tool-specific GUIs provide helpful information and allow running all analyses with independent settings. Furthermore, the batch script remembers the most recent user input and will pre-fill the previous choices by the user, helping to test settings and maintain track of chosen settings in case of incomplete runs. See the manual and worked examples for detailed instructions on how to use them. Users familiar with QCPA and MICA toolbox will find many GUIs similar to existing ones. However, the batch script features several custom-built GUIs to enable the flexible use of various user-defined processing steps.

### Workflow recommendations

The Batch GUI, manual and worked examples contain a suite of recommendations in addition to those in van den Berg et al. (2020a, b) and the empiricalimaging.com website that users might find informative. We recommend that the user validate the numerical accuracy of the batch script output on a small dataset before analysing large datasets. This also allows the user to confirm that the folder structure and auxiliary files have been arranged correctly. As processing large datasets can take many hours, days, or even weeks, we recommend that the user consider running sub-sections of larger datasets on different instances, such as multiple computers, servers, or cloud-based computing facilities. This will ultimately save time and prevent data loss while helping to detect faulty files in large datasets faster.

Like all approaches to visual modelling, image analysis with QCPA requires many different input choices by the user. Keeping a record of these choices is crucial for two reasons. First, it allows the user to keep track of settings, facilitating record-keeping and collaboration. Second, it provides for the publication of repeatable research. The latter is often an issue in visual modelling studies, as modelling choices are often poorly documented (White et al. 2015). To this end, the QCPA batch script keeps various detailed log files in the data output. These can easily be added to the supplementary information of any publications or uploaded/shared as part of the data.

## R script library

We provide a complimentary set of tailored R-scripts enabling the extraction and compilation of batch QCPA output data into .csv tables that can be used for downstream analysis. The efficient handling of QCPA batch output is crucial in using QCPA at larger scales, as various outputs are stored in files across a dedicated data set structure. The scripts and functions we provide here are formulated and commented on in a way that is aimed to facilitate modification by the user. These scripts and functions are intended to provide guidance for people with limited experience in managing large and complex datasets. However, these scripts and functions also aim to provide a starting point for more versed users to tailor their own data analysis pipelines. A detailed manual for using the R scripts can be found in the electronic Supplement or here: https://github.com/CaraConradsen/QCPA-r-script.

The R environment provided includes the following functionality:

1. Compiling CAA, VCA, BSA, and particle analysis data.
2. Compiling data from LEIA.
3. Compiling data from GabRat.
4. Compiling data from Particle Analysis and secondary data output.
5. Merging, data cleaning and reshaping various subsets of data.
6. Exploratory data visualisation and analysis.
7. Exporting compiled files.

## Worked examples

We provide detailed worked examples in the Supplement with test data and corresponding toolbox files, such as cone mapping models. These aim to highlight the functionality of the batch script and allow users to familiarise themselves with examples related to their needs. Instructions for the worked examples can be found in the electronic Supplement or here: https://github.com/cedricvandenberg/QCPA-batch-script.

In these worked examples, we showcase the use of the batch script across two of the most common uses of the QCPA. Namely, the consideration of an observer with and without UV-sensitive photoreceptors. These examples aim to familiarise the user with the required data preparations and provide specific examples of workflows that can be easily translated to a user’s project.

In the first example, we showcase the application of all modules in the script to investigate how a triggerfish (*Rhinecanthus aculeatus*) perceives two species of cryptic sea slugs (*Aphelodoris varia, Aplysia sp*.) from the east coast of Australia (Nelson Bay, New South Wales) photographed underwater (see the worked examples). The second example highlights the analysis of cryptic spiders (*Tamopsis brisbanensis*) found on eucalypt trees in Sydney, NSW (Australia) (see the worked examples), as seen by a bird with UV-sensitive photoreceptors.

## Discussion

We provide a user-friendly, open-source batch processing extension to QCPA, allowing for the semi-automated analysis of large datasets within the QCPA framework. This greatly facilitates the full-scale use of the analytical power of the framework as intended by its original design (van den Berg et al. 2020b). The open-source code of the script is presented and structured with the deliberate purpose of enabling users to create customised versions of the batch script, which might be more suitable to their intended purposes. The batch script has a detailed manual guiding users in preparing and analysing the image data. We further provide three worked examples and corresponding test data, covering contexts where users will likely use the script. Lastly, novice users are provided with a set of R-scripts aiding in the downstream analysis of the data generated by the batch script. These scripts are presented and structured in a way that facilitates users to customise them to their needs.

The script enables the semi-automated analysis of large image datasets, computing hundreds of colour pattern descriptors at multiple viewing distances for dozens, hundreds, or even thousands of observations. This has previously been impossible due to the time-consuming manual processing of images in QCPA. As QCPA and other colour pattern analyses using visual modelling approaches (e.g. Maia et al. 2019; Troscianko and Stevens 2015) merely relate to approximations of early-stage visual processing (e.g. Endler and Mielke 2005; Marr 2010; van den Berg et al. 2020a; van den Berg et al. 2020b; Vorobyev and Osorio 1998), considering the context and task-dependent relationships with and between a broad range of descriptors is critical (see van den Berg et al. 2022 for discussion). Thus, the presented batch script provides a crucial step towards such analytical approaches.

Unlike other image analysis tools available to visual ecologists, such as pavo (Maia et al. 2019), MICA is an ImageJ (Schneider et al. 2012) plugin written in Java (Arnold et al. 2005) and ImageJ batch script. It is intentionally tailored towards accessibility via graphical user interfaces (GUIs) rather than command line prompts. Unlike R software (R Core Team 2021) or Python (van Rossum 1995), frequently taught in undergraduate and HDR Biology degrees worldwide, Java or ImageJ batch script are not programming languages that biologists are readily familiar with. Therefore, writing scripts and macros for a complex network of GUI-guided tools in an unfamiliar language is a hurdle for most users of the QCPA framework seeking to batch-process large volumes of image data. This creates the potential for unintentional gatekeeping by researchers with access to programmers familiar with writing scripts in Java and an adequate understanding of the framework’s mechanisms. The result is a hidden custom of informally traded scripts, which undermines fast and equal access to methods in the field.

QCPA provides a conceptual approach towards combining visual modelling and colour pattern analyses. It does not claim a ‘perfect’ way of achieving its purpose. Rather, it provides an idea of how to combine different elements of vision modelling and colour pattern analysis, lending from and contributing to existing methods. Therefore, automating workflows and advancing customisability represent essential stepping stones towards the continued development and refinement of the framework and its contribution towards alternative methods. By providing an inviting, well-annotated, intuitive, open-access and open-source environment, we hope to facilitate the application of and, importantly, eventual modifications and additions to the framework and its combined use with other existing methodologies. Cumulatively, this round-table philosophy will not only advance the development and testing of methods in the field but, crucially, will facilitate the increased critical understanding of, and familiarity with, existing methodology among researchers in visual ecology. On a more tangible note, this script can easily be adjusted to enable the analysis of different data structures or include processing steps and analyses available in QCPA or the MICA toolbox currently not considered in the script. The R scripts and functions provided here provide useful guidance for users with variable experience in data wrangling. However, more extensive forms of such customised data extraction functions will provide an important step towards the dynamic use of large-scale QCPA output across multiple colour pattern analysis frameworks in the R environment, such as pavo.

### Supplementary Information

Below is the link to the electronic supplementary material.

Supplementary file1 (XLSX 29 KB)

## Data Availability

All data and supplementary materials are available at the respective repositories listed below.

## Supplementary data

Batch script ImageJ plugin, Batch script manual, worked examples guide: https://github.com/cedricvandenberg/QCPA-batch-script. R scripts and functions: https://github.com/CaraConradsen/QCPA-r-script. Test data for the worked examples (processed and unprocessed): https://doi.org/10.48610/3cdcc1f. The MICA toolbox and original QCPA, together with dedicated tutorials, guides and user forums can be found here: https://www.empiricalimaging.com/.

## Code availability

All code is available at the respective repositories listed above.
